# Mindfulness-based interventions in multiple sclerosis: beneficial effects of Tai Chi on balance, coordination, fatigue and depression

**DOI:** 10.1186/s12883-014-0165-4

**Published:** 2014-08-23

**Authors:** Janina M Burschka, Philipp M Keune, Ulrich Hofstadt-van Oy, Patrick Oschmann, Peter Kuhn

**Affiliations:** 1 Institute of Sports Science, University of Bayreuth, Bayreuth 95440, Germany; 2 Klinikum Bayreuth GmbH, Betriebsstätte Hohe Warte, Department of Neurology, Hohe Warte 8, Bayreuth 95445, Germany; 3 Department of Physiological Psychology, Otto-Friedrich-University, Bamberg, Germany

**Keywords:** Multiple Sclerosis, Tai Chi, Mindfulness, Balance, Depression

## Abstract

**Background:**

Patients suffering from Multiple Sclerosis (MS) experience a wide array of symptoms, including balance problems, mobility impairment, fatigue and depression. Physical exercise has recently been acknowledged as a treatment option complementary to medication. However, information regarding putative effects of structured exercise programs on neurological symptoms is sparse. Tai Chi, a Chinese martial art incorporating physical exercise and mindfulness training, has been shown to yield health benefits in various neurological groups. It seems particularly suitable for patients with motoric deficits as it challenges coordination and balance. The purpose of the current study was to explore the therapeutic value of structured Tai Chi training for coordination, balance, fatigue and depression in mildly disabled MS patients.

**Methods:**

A sample of 32 MS patients (Expanded Disability Status Scale, EDSS < 5) was examined. A structured Tai Chi course was devised and a Tai Chi group participated in two weekly sessions of 90 minutes duration for six months, while a comparison group received treatment as usual (TAU). Both groups were examined prior to and following the six-months interval with regards to balance and coordination performance as well as measures of fatigue, depression and life satisfaction.

**Results:**

Following the intervention, the Tai Chi group showed significant, consistent improvements in balance, coordination, and depression, relative to the TAU group (range of effect-sizes: partial η^2^ = 0.16 – 0.20). Additionally, life satisfaction improved (partial η^2^ = 0.31). Fatigue deteriorated in the comparison group, whereas it remained relatively stable in the Tai Chi group (partial η^2^ = 0.24).

**Conclusions:**

The consistent pattern of results confirms that Tai Chi holds therapeutic potential for MS patients. Further research is needed to determine underlying working mechanisms, and to verify the results in a larger sample and different MS subgroups.

## Background

Multiple Sclerosis (MS) is the most common neurologic disease of young adults [1]. It is characterized by chronic inflammatory processes affecting the central nervous system, causing neurodegeneration and axonal damage [2]. While the early phase of the disease involves temporary neurologic dysfunctions, the disease course leads to progressive accumulation of disability. In this context, patients suffering from MS may experience a wide array of symptoms, including balance problems, mobility impairment, fatigue and depression [2],[3]. Common medical treatment comprises basic immune-modulating medication, cortisol therapy to counter active inflammation during relapses, as well as symptomatic therapy [4].

### Physical therapy for multiple sclerosis

Physical exercise has recently been acknowledged as an essential part of MS therapy [5]. This represents an important extension of treatment options, since traditionally MS patients had been advised not to engage in physical activity [6]. There is a growing body of evidence which suggests that exercise is beneficial for MS patients in various domains including muscular strength and aerobic capacity, mobility, mood, fatigue and health-related quality of life [4],[7]–[10]. Additionally, physical exercise may yield potentially adaptive immune-modulating effects [11],[12]. Recent findings also suggest that repeated physical exercise may foster neuroplasticity, affecting neurotrophic and neuroprotective mechanisms [13]. However, most MS patients remain physically inactive and information regarding choice, dose and effects of specific exercise programs is sparse [9]. Ellis et al. [14] suggest the inclusion of behavioral interventions into standard MS management, and propose a shift from a rehabilitative model to a preventive approach of physical therapy (for comparison see also Motl et al. [15]). As there is considerable inter-individual variation in the course of symptoms in MS, individually tailored exercise programs may be regarded as most appropriate.

### Mindfulness and its clinical applications

Recently, such programs have successfully been implemented in combination with mindfulness based interventions (for recent review see Simpson et al. [16]). The term mindfulness is rooted in Buddhist philosophy [17]. Its key element is the attempt to focus on the present moment experience while maintaining and open, non-judgemental attitude [18],[19]. Mindfulness has repeatedly been incorporated into secular clinical interventions. In this context, mindfulness training was shown to yield beneficial effects in a variety of conditions including chronic pain, fibromyalgia, psoriasis, as well as depressive and eating disorders, and to attenuate maladaptive cognitive patterns [20]–[26]. Further studies indicate that mindfulness training may yield adaptive neurophysiologic effects, including altered immune functioning [27]–[29].

**Figure 1:**
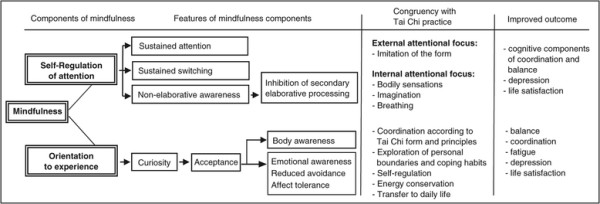
**Conceptual outline of the two**-**component model of mindfulness,adapted from Bishop et al. (2004) and its relevance for Tai Chi practice.**

While the exact working mechanisms of mindfulness continue to be explored, integrative models have been devised. In their two-component model of mindfulness, Bishop et al. [30] defined an attentional and a motivational component. A conceptual outline of this model is provided in Figure 1. According to the authors, self-regulated attention involves focusing on experience in the here and now. The resulting ability to sustain attention and switch its focus intentionally is assumed to enable non-elaborative awareness. The latter is characterized by observing, acknowledging and releasing arising thoughts, sensations and emotions without further, secondary cognitive elaboration. Since attentional resources are limited, non-elaborative awareness increases access to information on current experience, as attentional capacity is not allocated to further elaborative processing [31]. In mindfulness exercises, commonly the breath is used as an object of orientation to return back to the present moment experience.

The second component, orientation to experience, implies a curious and accepting attitude towards the stream of consciousness (*beginner*’*s mind*, *see* Bishop et al. [30]). This observant, non-judging perspective and the open receptivity to new experience are assumed to reduce avoidant behavior patterns [29]. In this context, particularly the familiarisation with distressing emotions is believed to increase affect tolerance. Moreover, body awareness and emotional awareness are fostered.

### Tai Chi training and mindfulness

To date, there are only few structured interventions which incorporate both, physical and mindfulness exercises for MS patients. Grossman et al. [32] implemented a structured eight-week mindfulness-based intervention including Yoga exercises and observed improved quality of life, as well as depression and fatigue. Complementary to such pioneering work, we suggest that Tai Chi may be particularly suited to integrate physical and mindfulness training in MS. Tai Chi is a Chinese martial art, representing a multicomponent intervention, addressing musculoskeletal strength, flexibility, and mindfulness [33],[34].

It should be noted that the concept of mindfulness is compatible with contents of Tai Chi practice. As illustrated in Figure 1, its first component, regulation of attention, is addressed by Tai Chi practice, as Tai Chi involves purposeful direction of attention. Attention needs to be directed during the process of imitating a choreography (called *form*) as demonstrated by the instructor. To this end, it is necessary to constantly switch attention between exteroception (observing the instructor and the own body) and interoception (bodily sensations involving proprioception, balance, breathing). During exercises, attention is also directed at the Tai Chi principles (see Additional file 1: Supplement 1), which require a high amount of resources and receptivity to interoceptive information, fostering body awareness.

The second component of mindfulness is addressed as Tai Chi training implies the maintenance of a curious and open attitude. The perpetual aim in Tai Chi practice is a friendly attitude towards oneself and others, respecting the own person and the own abilities, without judgement or competition (see Additional file 1: Supplement 1). Non-elaborative awareness is a prerequisite to stay on track while playing the form. During this process, personal boundaries such as limits of stability, flexibility and endurance, as well as habits to cope with these limitations are explored, fostering body awareness. Emotional awareness is developed by consciously observing and accepting arising emotions during Tai Chi practice (e.g. fear of embarrassment, fear of falling, self-criticism, motivational issues).

### Purpose of the current work: structured Tai Chi training for MS patients

Tai Chi has been shown to yield health benefits in various neurological groups, including Parkinson’s Disease [35], Fibromyalgia [36], chronic stroke [37], and peripheral neuropathy [38]. Currently there are three studies available on Tai Chi and MS. Husted et al. [39] reported improvements in walking distance, hamstring flexibility and psychological well-being, following a Tai Chi intervention. Mills et al. [40],[41] observed improvements in balance and symptom management. Finally, Tavee et al. [42] reported improvements in perceived physical and mental health, as well as pain, but not in mobility.

In the indicated studies, the intervention duration did not exceed 2 months. This is noteworthy, since the benefits of Tai Chi practice are believed to increase with time [43]. As a consequence, short intervention periods could lead to an underestimation of the full potential of Tai Chi [43]. Compatible with this assumption, recent findings suggest that the effect of mindfulness training on cognitive control and its underlying neural mechanisms is modulated by practice adherence [44].

For the current study, a standardized Tai Chi program was designed, details of which are made available to other research groups (Additional file 1: Supplement 1). The purpose of this standardized intervention was to provide a basis for a structured empirical evaluation of putative effects of Tai Chi in MS.

The goal of the current study was to explore the effects of this intervention program on balance, fatigue, depression and quality of life in mildly disabled MS patients. Furthermore, the intention was to examine the safety and feasibility of this intervention for MS patients.

## Methods

### Study design and participants

The study was designed as a two-arm trial to examine the effects of a six-month mindfulness-based Tai Chi intervention versus treatment as usual (TAU). It was approved by the ethics committee of the Bavarian Medical Association, Germany, and all participants provided written informed consent. Participants were recruited from the Department of Neurology, Klinikum Bayreuth. Invitations were given orally or via mail to patients who were or had been in out-patient care. Additionally, study invitations were distributed via local support groups. Information about clinical characteristics was extracted from patients’ files held by the Department of Neurology. Inclusion criteria were a diagnosis of any MS type, being able to walk without a walking aid, an Expanded Disability Status Scale (EDSS) score < 5, and being relapse-free for the past four weeks. Severe cognitive impairment which would interfere with the ability to take part in weekly Tai Chi classes was ruled out, based on reports of the neurological examinations in patients’ files.

Between December 2010 and November 2011, the files of a total of 400 MS patients were screened for eligibility criteria. Since the site of the study (Klinikum Bayreuth) is located in a rural area of Germany, a substantial portion of these candidates (approximately 250) were discarded beforehand due to the distance of their home to the study site, which made a weekly appearance impossible. Out of the remaining candidates, 38 met inclusion criteria and were willing to participate in the study. Outcome measures were assessed during a patient visit at baseline and following an interval of six months. In this context, all patients participated in assessments addressing balance, fatigue, depression, and quality of life. Potential alterations in balance and coordination were defined as primary endpoints, alterations in fatigue, depression and quality of life as secondary endpoints of the study. During the six-months interval between pre (baseline) and post assessments, 15 patients received structured Tai Chi training (Tai Chi group), and 17 patients received treatment as usual (TAU group), i.e. they were instructed to consult their medical professionals as they usually would. In a pilot phase, implemented with the intention to raise awareness about the possibility to participate in Tai Chi courses in the study centre, members of the latter group had previously taken part in Tai Chi classes. However, during the six-months interval, members of this group did not participate in a structured intervention. Due to the length of the intervention and to enable patients to participate regularly, group assignment occurred based on patients’ availability for the weekday on which the Tai Chi course took place.

Six Patients from the Tai Chi group withdrew from the study due to time issues (N = 5) and health problems (N = 1) and were lost to follow-up. Consequently a total of 32 patients was included in the final analysis (Tai Chi N = 15; TAU N = 17). As indicated in Table 1, there were no significant differences with regards to basic demographics between the Tai Chi group and the TAU group. Further, there were no clinical differences regarding MS course, disease duration and MS treatment. However, the EDSS was elevated in the TAU group (range: 1–4.5, median = 4), relative to the Tai Chi group (range: 1–4, median = 2). Adherence varied between 15 and 44 (median = 30) attended classes out of 50 classes offered in total.

**Table 1:** **Demographics**, **clinical information**, **health behavior**

	**TAU**		**Tai Chi**		**Statistic**	**p-value**
	**(n** **=** **17)**		**(n** **=** **15)**			
**Demographics**						
Age M (SD)	43.6	(8.0)	42.6	(9.4)	0.32^a^	0.753
Female sex, n (%)	12	(71)	10	(67)	0.06^b^	0.811
**Health Behavior**						
Tobacco users, n (%)	2	(12)	3	(20)	0.42^b^	0.645
Body Mass Index, M (SD)	25.5	(5.5)	24.2	(3.7)	0.79^a^	0.438
Phys. activity/week, n (%)						
< 1x	6	(35)	2	(13)	0.17^b^	0.338
1-2x	6	(35)	8	(53)		
> 3x	5	(30)	5	(33)		
**Clinical Information**						
MS course, n (%)						
Relapsing-remitting	13	(77)	14	(93)	4.93^b^	0.085
Secondary progressive	4	(24)	0	(0)		
Clinically isolated syndrome	0	(0)	1	(7)		
MS Duration in years, M (SD)	7.8	(6.8)	6.0	(4.7)	0.86^a^	0.395
MS treatment, n (%)						
Yes	16	(94)	12	(80)	0.45^b^	0.228

^a^t-test.

^b^chi square test.

Note: M = mean, SD = standard deviation, TAU = treatment as usual.

### Assessment of balance and coordination

An established balance test, comprising 14 tasks with an increasing level of difficulty, including both static and dynamic balance was utilized [45]. It included a series of one leg stances in different conditions as well as walking across a wooden board in different conditions (forwards, backwards, including turns). The test was previously shown to display sufficient metric qualities (test-retest reliability: r = .78, Chronbach’s alpha = .92) [45]. In this context, its convergent validity was also shown referring to posturographic measures [45].

Additionally, a coordination test was implemented, comprising 10 tasks with an increasing level of difficulty [45]. As was the case for the balance test, the coordination test was previously shown to involve sufficient metric properties (test-retest reliability: r = .60, Chronbach’s alpha = .72) [45].

In both tests, each task achieved equalled one point, accumulating to a maximum of 14 points in the balance test and 10 points in the coordination test. A detailed description of both tests is provided in Additional file 2: Supplement 2. To our knowledge, the tests have not been used in MS previously. However, they were developed for functional evaluation of patients during neurologic rehabilitation [45]. Since the tests were designed to cover a broad spectrum of ability/disability within ambulatory patients, they may be regarded as particularly suitable for MS patients with considerable variance in motoric deficits.

### Assessment of depression, fatigue and life satisfaction

Additionally, self-reports measures in the domains depression, fatigue and life satisfaction were included. For the assessment of depressive symptoms, a 15-item questionnaire was used (Allgemeine Depressionskala, ADS-K; English: Center for Epidemiological Studies Depression Scale, CES-D); [46]), which addresses the severity of depressive symptoms during the last two weeks. Items are rated on a scale from 0–3 and the sum of all items represents the depression parameter. Further, the Fatigue Scale of Motor and Cognitive Functions (FSMC [47]) was administered, which consists of 20 items, scored on a scale from 1–5, and accumulating to a maximum score of 80 points. The Questionnaire of Life Satisfaction (QLS [48]) consists of 6 domains, including health, finances, leisure time, self, sexuality and friends, comprising 7 items each, on a 1–7 rating scale, accumulating to a maximum score of 420 points.

### Tai Chi intervention

A structured, compact Tai Chi program was developed based on the Yang-style 10-form (see Additional file 1: Supplement 1 for details and Martin [49] or Sinclair [50] for a video link). Exercises were structured so that during each session of the course, the same essential elements were repeated. The advantage of this design was that patients who missed out on an occasion could rejoin the training without difficulties. Further, continuously repeating the exercises may be regarded as supportive for a long-term learning process and automaticity, potentially fostering neuroplasticity [13]. The intervention was centre-based and did not include home assignments. The Tai Chi instructor was a highly trained exercise therapist with 4 years of Tai Chi experience. No classes were cancelled and no adverse events were noted.

Throughout the intervention period of six months, weekly sessions of 90 minutes duration took place. During the first month of the course, the Tai Chi form was played 4–5 times per session. A break of a few minutes during which participants could relax and ask questions occurred after each completion of the form. During the consecutive 5 months the form was played 6–8 times per session. In this case, breaks usually occurred after two completions of the form, respectively, albeit they were not always necessary or enforced. The increase in repetitions following the first month was attributable to arising automaticity. In context of the relation between Tai Chi training and mindfulness (Figure 1), this arising automaticity may be associated with an attentional shift from an external focus (imitating the form) to an internal focus (e.g. bodily sensations, breathing). On the occasion with the highest amount of repetitions without a break, 8 repetitions were completed. In general, the instructor organized the class with respect to the energy level and current capability of participants.

### Statistical analysis

Scores on all parameters were normally distributed according to Saphiro-Wilk tests (all *p*-values > 0.05). Differences in outcome parameters between the Tai Chi group and the TAU group were assessed by a two-way repeated measures ANOVA with the within-subjects factor Time (pre vs. post) and the between-subjects factor Group (Tai Chi vs. TAU), separately for each parameter. Post-hoc comparisons were conducted via Bonferroni-corrected two-sided t-tests.

To ensure systematic progression of variance across pre and post measures, in a secondary analysis, test-retest reliability was determined. To this end, Pearson correlations were computed for each test parameter between values obtained at pre and post assessments.

## Results

### Balance and coordination

**Figure 2:**
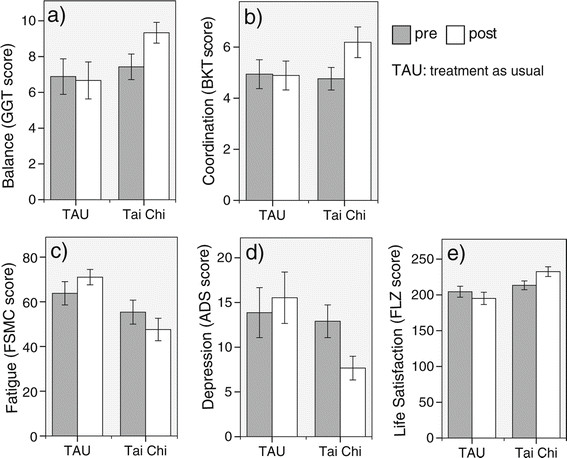
**Mean scores on outcome parameters compared between the Tai Chi and the TAU group.** Error bars represent standard errors. See Methods section for a detailed description and references of each test.

In case of balance performance, a significant Time by Group interaction emerged [F (1,30) = 5.70, p < 0.05, partial η^2^ = 0.16]. Post-hoc comparisons revealed that performance in the Tai Chi group improved, while it remained relatively stable in the TAU group (Figure 2a, Table 2). For coordination, there was a significant main effect of Time [F (1,30) = 4.89, p < 0.05, partial η^2^ = 0.14] and a significant Time by Group interaction [F (1,30) = 6.57, p < 0.05, partial η^2^ = 0.18]. Post-hoc comparisons indicated that the increase in coordination performance scores was significant in the Tai Chi group whereas scores remained relatively stable in the TAU group (Figure 2b, Table 2).

**Table 2:** Differences in mean scores on outcome parameters in the Tai Chi group and the TAU group

	**TAU(N** **=** **17)**					**Tai Chi(N** **=** **15)**				
	**Pre**		**Post**		**Statistic**	**Pre**		**Post**		**Statistic**
	**M**	**SD**	**M**	**SD**	**p**	**M**	**SD**	**M**	**SD**	**p**
**Balance**	6.88	4.09	6.53	4.49	0.439	8.00	2.83	9.33	2.26	0.031
**Coordination**	4.94	2.33	4.82	2.46	0.814	5.00	1.89	6.60	1.80	0.003
**Fatigue**	63.79	19.55	70.47	14.04	0.025	51.23	22.55	47.6	19.54	0.182
**Depression**	13.87	10.82	16.13	11.99	0.951	12.21	6.66	7.67	5.12	0.007
**Lifesatisfaction**	204.46	27.77	193.81	36.2	0.290	215.77	25.55	232.57	25.62	0.012

Note: M = mean; SD = standard deviation; TAU = treatment as usual.

### Depression, fatigue and life-satisfaction

In case of depression, a significant main effect of Time [F (1,27) = 6.61, p < 0.05, partial η^2^ = 0.19] and a significant Time by Group interaction emerged [F (1,27) = 6.55, p < 0.05, partial η^2^ = 0.20]. Post-hoc comparisons revealed that depression scores significantly decreased in the Tai Chi group, whereas they remained relatively stable in the TAU group (Figure 2d, Table 2).

For Fatigue, a significant Time by Group interaction [F (1,25) = 7.83, p = 0.01, partial η^2^ = 0.24] and a significant main effect of Group emerged [F (1,25) = 5.91, p < 0.05, partial η^2^ = 0.19]. Post-hoc comparisons indicated a significant increase in fatigue scores in the TAU group from pre to post assessments. Scores in the Tai Chi group remained relatively stable. At pre-treatment assessments the groups did not differ significantly in fatigue. In contrast, fatigue differed following the six-months interval (Figure 2c, Table 2).

In case of life satisfaction, a significant Time by Group interaction [F (1,24) = 8.64, p < 0.01, partial η^2^ = 0.27] and a significant main effect Group emerged [F (1,24) = 8.64, p < 0.01, partial η^2^ = 0.19]. Post-hoc comparisons indicated a significant rise in the life-satisfaction score in the Tai Chi group relative to the TAU group. At pre-treatment assessments the groups did not differ significantly in life satisfaction. In contrast, life satisfaction differed following the six-months interval (Figure 2e, Table 2).

### Test-retest reliability

In consideration of the relatively small sample size, estimates of test-retest reliability were computed to derive information on the reliability of all implemented measures. This analysis was implemented separately for the Tai Chi and the TAU group, as well as for both groups combined, and tested whether the progression of variance of each parameter was systematic across assessments [51],[52]. Test-retest reliability was reasonable for each parameter in both groups (Table 3; range of Pearson r: .56 - .92).

**Table 3:** **Test**-**retest reliability**

	**Tai Chi & TAU**		**Tai Chi**		**TAU**	
**Parameter**	**r**	**p**	**r**	**p**	**r**	**p**
**Balance**	0.835	0.000	0.660	0.007	0.913	0.000
**Coordination**	0.573	0.001	0.565	0.028	0.642	0.005
**Fatigue**	0.877	0.000	0.917	0.000	0.878	0.000
**Depression**	0.822	0.000	0.653	0.011	0.936	0.000
**Life satisfaction**	0.697	0.000	0.660	0.014	0.757	0.003

Test-retest reliability as determined by Pearson correlations between values obtained at pre and post assessments (Tai Chi group N = 15, TAU group N = 17). See Methods section for a detailed description of each test parameter.

## Discussion

Physical exercise has recently been acknowledged as an essential part of MS therapy [5]. However, information with regards to putative effects of structured exercise programs on neurological symptoms is sparse. Similarly, mindfulness training, which is known to be beneficial for various clinical groups [53],[27], was suggested to increase quality of life in MS patients [54]. Nevertheless, to date the body of studies on this topic remains limited [16]. Previous work has indicated that Tai Chi, which incorporates a combination of physical exercises and mindfulness training, might yield health benefits in MS [16],[39]–[42]. In the current study, the therapeutic value of a newly devised structured Tai Chi course (Additional file 1: Supplement 1) for coordination, balance, fatigue and depression in mildly disabled MS patients was examined.

The obtained results are in accordance with previous suggestions and extend the body of related literature [55],[56],[10],[16],[4]. During the Tai Chi intervention, objective parameters including balance and coordination (i.e. the primary study endpoints) as well as subjective measures of depression and life satisfaction (secondary endpoints) improved. In contrast, these measures were not altered in the TAU group. Additionally, a maintenance effect on fatigue was observed, as fatigue in the Tai Chi group remained relatively stable whereas it deteriorated in the TAU group. In sum, this consistent pattern of beneficial alterations in the examined outcome parameters provides support for the utility of structured Tai Chi training in the context of MS treatment. While the exact working mechanisms remain to be explored, the current findings allow the suggestion that Tai Chi enhances balance performance and coordination and may exert beneficial effects on mood and fatigue. Observed compliance rates indicated that the implemented Tai Chi form (10-form) was appropriate in the context of a structured intervention of six months duration. We recommend using the 10-form in future studies as it is a well-known standard form and provide extensive information on the intervention in Additional file 1: Supplement 1.

### Limitations and future directions

While our results extend the current literature on beneficial effects of mindfulness training and physical exercise in MS patients, they need to be interpreted in the context of several limitations. On the one hand, the length of the intervention made it necessary to ensure that a sufficient number of patients could participate in the Tai Chi course regularly. Therefore, assignment to Tai Chi and TAU groups occurred based on patients’ weekday preference. The lack of formal random assignment is to be regarded as a major limitation of the current work. Moreover, group assignment based on patients’ weekday preference represents a selection bias and may have been affected by patients’ general motivation. In this context, it is also necessary to point out that while some of the clinical characteristics were similar in both groups (MS-subtype, disease duration, medical treatment), the EDSS score was higher in the TAU group than in the Tai Chi group. Despite its relatively broad classification system, the EDSS is a widely used and accepted tool for monitoring overall disease progression in MS patients [57]. Even though functional domains reflected by the EDSS are not congruent with all parameters implemented in the current work, it cannot be ruled out that the difference in EDSS scores of the two groups affected the obtained results. It also needs to be considered that only a small sample was examined and that the intervention and evaluation procedures were implemented by the same person (JB), involving a potential bias. Moreover, the dose of Tai Chi training was not recorded in detail. However, the total dose of Tai Chi cannot easily be measured as it depends on multiple components and personal commitment of the participants.

On the other hand, it is noteworthy that the pattern of beneficial effects was consistent across outcome variables and that patients’ compliance was sufficient. Moreover, despite the small sample size and the relatively long intervention period of six months, test-retest reliability of all test parameters was sufficient. This is particularly important since the implemented tests were not instruments which are widely used in the MS literature. Consequently, it could be argued that they might have involved low validity in MS patients. Contradicting this assumption, the observation of sufficient test-retest reliability of all test parameters in the current work indicates that rank ordering of subjects’ values in outcome parameters changed systematically during the six months period. Hence, the progression of variance was systematic and employed measures provided reliable information. We suggest that it is warranted for future studies to verify results of the current work using a randomized design, a larger sample and different MS subgroups. Such future studies may also include further comparison groups, besides TAU, especially in consideration of non-specific effects which might manifest in improved depression symptoms and quality of life due to social support, as well as self-efficacy.

Analyzing different components of Tai Chi interventions, such as motion, mindfulness and breathing, may help to form theories about underlying working mechanisms. In this context, the effects of Tai Chi training on neural plasticity with respect to motor and sensory abilities in MS patients seem worth investigating as recent findings showed alterations of cortical representations of interoceptive attention following mindfulness meditation [58]. Improved interoception may also enhance the quality of feedback loops during coordination, enhancing confidence and reliability of movement. Apart from this, it has been shown that breathing practices by themselves have beneficial autonomic regulatory effects [59]. This is of particular interest in stress-exacerbated diseases like MS, as it has been reported that stress management therapy seems to reduce inflammation and enhance neuroprotection [60]. Based on the working mechanisms of different components, possible interactions and synergetic effects need to be explored. This understanding might provide the opportunity of creating clear recommendations on which way of practicing Tai Chi is likely to unfold most beneficial effects [34].

## Conclusions

The Tai Chi 10-form is safe and feasible for a six-months intervention period with MS patients. Tai Chi may have beneficial effects on balance, coordination and psychological well-being in patients with MS.

## Competing interests

The authors declare that they have no competing interests. The study was supported by Novartis Pharma GmbH. The corresponding author received an expense allowance for presenting the study on a symposium of Novartis Pharma GmbH. This publication was funded by the German Research Foundation (DFG) and the University of Bayreuth in the funding program Open Access Publishing.

## Authors’ contributions

JB conceived of the study, participated in its design, coordination and data acquisition, held all Tai Chi classes and drafted the manuscript. PKe performed the statistical analysis and helped to draft the manuscript. UH supported the patient recruitment and clinical assessments. PO participated in the design of the study, supervised the study and reviewed the manuscript. PKu participated in the design of the study, co-conceptualized and supervised the Tai Chi intervention and revised the manuscript. All authors read and approved the final manuscript.

## Additional files

## Supplementary Material

Additional file 1: Supplement 1. Click here for file

Additional file 2: Supplement 2. Click here for file
