# Supplementary material for: Mindfulness-based interventions in multiple sclerosis: beneficial effects of Tai Chi on balance, coordination, fatigue and depression
Source: BMC Neurol. 2014 Aug 23;14:165. doi: 10.1186/s12883-014-0165-4 (PMC4236646; doi:10.1186/s12883-014-0165-4)
Supplement: Additional file 1: — Supplement 1. [file s12883-014-0165-4-S1.doc]

Supplement 1

The 10-form Tai Chi protocol is a simple concept, adaptable to the abilities and needs of participants. The primary aim of each lesson is to provide the participants with an opportunity to employ in a self-determined exploration of the body and its abilities. Each lesson consists of four elements: warm-up, form, consolidation, cool-down. The *warm-up phase* includes two weight shifting exercises and is split in a time for conversation and a time in silence. Any further exercises are to be excluded to keep the intervention as simple as possible. *The complete 10-form* is practiced several times each lesson. The number of repetitions varies depending on the mood of the participants. There may be several repetitions without a break in between. The time to complete the form once takes about 5 to 6 minutes. Additionally, each lesson contains a *technique consolidation phase* on specific elements of the form, based on the questions and needs of the participants. The lessons are concluded with a *cool-down phase*, comprising breathing and relaxation techniques which focus on body awareness and abdominal breathing in a sitting or lying position. This curricular concept allows for a ritualized course of Tai Chi training with a focus on the Tai Chi movements and principles. Included principles are body-alignment, moving the body slowly as a whole, staying balanced and aware of the body’s centre of gravity as well as of the body’s connection to the ground, awareness to weight-shifting and not falling into the step, mindful movement, relaxation and abdominal breathing. The training may be accompanied by music. We used the audiobook [*Shaolin Qi Gong - Energy in Motion (Energie in Bewegung*](http://www.amazon.de/Shaolin-Qi-Gong-CD-Bewegung/dp/386728024X/ref=sr_1_3?ie=UTF8&qid=1392472727&sr=8-3&keywords=shaolin+qi+gong)) by Sayama and Shi Xinggui (2007).

1. Warm-up (10-20min)
   1. The first exercise comprises rhythmic weight shifting from one leg to the other while swinging the relaxed arms from one side to the other via a rotation in the trunk and hip. The heel may be lifted from the floor when the body is turning away from it. The instructor should coach the participants regarding physiologic movement of the knees if necessary. During this activity, there is room for conversation.
   2. The second exercise consists in shifting the body’s weight to different directions in a relaxed and upright, standing position, feet hip-width apart. First, awareness is directed on a grounded, balanced and upright position, relaxed shoulders and comfort. Second, participants start slowly shifting their weight in different directions like a tree in the wind. They are encouraged to shifting within the comfort zone as well as beyond their comfort zone to explore their limits of stability. Third, the movements are reduced until the participants reach their centre, standing almost still.
2. 10-Form
   The 10-form is played in a symmetric way. While the figures commencement, cross hands and conclusion are played once, the remaining figures are played twice in opposite directions. The 10 figures are: commencement, reverse reeling forearms, brush knee and push, part the wild horse's mane, wave hands like clouds, rooster stands on one leg, kick with heel, grasp the peacock's tail, cross hands, conclusion. Depending on the way of counting, the 10-form is sometimes also called the 8-form (not counting the commencement and the conclusion). A video of the form can be found here: http://www.youtube.com/watch?v=P4Z4sQNQtJM or here http://www.youtube.com/watch?v=n_nfD5O15bk.
3. Consolidation
   The consolidation phase is used to pick specific elements from the form or principles for more detailed explanation and practice. The content of the consolidation phase is variable, depending on the participants’ questions and needs. The aim of this phase is to help the participants to get more comfortable with the Tai Chi form. The accuracy of the Tai Chi movements according to the standard version are supported but not demanded. During the first two months of the intervention, the one leg stances are practiced with the free foot touching the ground with the toes (rooster stands on one leg) or heel (kick with heel). After two months, the teacher starts demonstrating the single leg stance version. Participants are encouraged to choose their preferred way of playing the positions. The teacher emphasizes that the relaxed stability of the position (even with the help of the free foot) is more important than barely managing a single leg stance.
4. Cool-down (15min)

During the cool-down phase, the attention is directed to the inside. The aim is mindful body awareness and abdominal breathing. First, the attention is directed to the body’s contact to the ground and a feeling of being supported. Trusting this support, participants let go of weight and tension. Second, the attention is consecutively drawn to the breath––how and where it can be felt. Third, the hands are placed on the belly and try to feel the breathing. Forth, the incoming air is directed towards the hands to motivate abdominal breathing. Fifth, active engagement to breathing turns to simple awareness of the flowing movements of breathing and the alternation of expansion and release. Sixth, the attention is directed to the ground and further into the room, leading the participants back to the outside world. Seven, there is time for gentle movement, stretching and yawning. Eighth, in an upright position, the participants wake themselves up by tapping their body.
