# Supplementary material for: Mindfulness-based interventions in multiple sclerosis: beneficial effects of Tai Chi on balance, coordination, fatigue and depression
Source: BMC Neurol. 2014 Aug 23;14:165. doi: 10.1186/s12883-014-0165-4 (PMC4236646; doi:10.1186/s12883-014-0165-4)
Supplement: Additional file 2: — Supplement 2. [file s12883-014-0165-4-S2.doc]

Supplement 2

| **Balance Test Description[[1]](#footnote-2)** |  |
| --- | --- |
| 1. aim | Quantitative measure of balance ability |
| 1. tools | 1 balance beam (10cm in width, 4m in length, 1cm in height)  2 cones  1 volleyball |
| 1. content | 14 Items, rated dichotomously (1 = achieved task, 0 = failed task) |
| 1. course | test abortion after two consecutive fails |

| **Coordination Test Description** |  |
| --- | --- |
| 1. aim | Quantitative measure of balance ability |
| 1. tools | 1 balance beam (10cm in width, 4m in length, 1cm in height)  2 cones  1 volleyball  1 gymnastics ball  1 gymnastics stick  3 target marks (20cm in diameter, 70cm apart, two in the height of 1.18m, one in the height of 1.74m), 1 target mark (1m in diameter, in the height of 3m) |
| 1. content | 10 Items, rated dichotomously (1 = achieved task, 0 = failed task) |
| 1. course | test abortion after two consecutive fails |

Conduction the balance test and the coordination test combined

| Coordination | Balance | # | task | description | |
| --- | --- | --- | --- | --- | --- |
|  |  |  | The Test is comprised of 24 tasks in a rising level of difficulty. Try to complete as many tasks as possible. It is important that you keep up your concentration even when you failed to complete a task. The test is going to take this course: We do one task after the other. At first, I’m going to demonstrate the task for you and explain its rules. While I’m doing that you are not allowed to try the task yourself, instead you just watch and listen. When it’s your turn, I’m going to repeat the aim of the task for you. Consecutively, you try to complete the task. You only have one single try for each of the tasks. | | |
|  | x | 1 | One leg stance | | As soon as you’re ready, you lift one knee up to the front.[[2]](#footnote-3) Your free foot mustn’t touch your standing leg and should be placed between the ankle and knee of you standing leg. The aim of this task is to keep standing on one leg for 15 seconds (s) without leaving the initial position of your standing foot. The time starts as soon as your foot left the ground. |
|  | x | 2 | One leg stance, moving the free leg forth and back | | As soon as you’re ready, you start swinging one leg forth and back. The aim of this task is to stand 15s on one leg, while swinging the other. Your standing foot may not leave its initial position during the task. The time starts as soon as your foot left the ground. |
|  | x | 3 | One leg stance after 360° turn | | As soon as you’re ready, you turn 360 degrees. Right after turning, you lift one knee up to the front. Your free foot mustn’t touch your standing leg and should be placed between the ankle and knee of you standing leg. The aim of this task is to keep standing on one leg for 15s without leaving the initial position of your standing foot. The time starts as soon as you completed your turn and your foot left the ground. |
| x | x | 4 | Jumping Jack | | Stand on this line with both feet touching each other and your arms relaxed beside your body. From this position, you jump into a wider stance and lift your arms sideways into the air simultaneously. Consecutively, you jump back into your initial position. The aim of this task is to do 5 jumping jacks, touching the line with at least one foot each time you land. As soon as you leave the line with both feet, you fail the task. |
|  | x | 5 | One leg stance with eyes closed | | As soon as you’re ready, you lift one knee up to the front. Your free foot mustn’t touch your standing leg and should be placed between the ankle and knee of you standing leg. Then you close your eyes. The aim of this task is to keep standing on one leg for 15s without leaving the initial position of your standing foot. The time starts as soon as you closed your eyes. |
|  | x | 6 | One leg stance with eyes closed after 360° turn | | As soon as you’re ready, you turn 360 degrees. Right after turning, you lift one knee up to the front. Your free foot mustn’t touch your standing leg and should be placed between the ankle and knee of you standing leg. Then you close your eyes. The aim of this task is to keep standing on one leg for 15s without leaving the initial position of your standing foot. The time starts as soon as you closed your eyes. |
|  | x | 7 | One leg stance, moving the free leg forth and back with eyes closed | | As soon as you’re ready, you start swinging one leg forth and back. Then you close your eyes. The aim of this task is to stand 15s on one leg, while swinging the other. Your standing foot may not leave its initial position during the task. The time starts as soon as you closed your eyes |
| x | x | 8 | One leg stance, drawing the figure 8 around two cones | | With one foot, you step between these cones, your feet should be about 15cm apart. The aim of this task is to draw the figure 8 around these cones with your leg. Keep this leg as straight as possible. You start drawing the figure 8 moving it sideways and then so the front around the front cone. After that, you move your leg between the cones and around the back cone and place it on its initial position between the cones. Try not to touch the cones on your way. Please do the task once with your eyes open and a second time with your eyes closed. If you don’t feel comfortable closing your eyes, you may refrain from trying the task with your eyes closed. |
| x |  | 9 | Touch three targets with a stick | | You stand sideways to the wall with your dominant hand facing the wall holding the stick. The aim of this task is to touch these 3 target marks consecutively with the stick. At first, you touch them with your eyes open. After that, you close your eyes and try to hit them again. Please do this task slowly and concentrated. |
| x |  | 10 | Moving pattern along the wall | | The aim of this task is to move sideways to the right along the wall in a specific pattern. In the initial position, you are standing face to the wall with your hands touching the wall at about the height of your shoulders. Your feet are touching each other. Your hands are touching each other. Then you start the movement with your right foot and your left hand. Your right foot takes a step to the right. Simultaneously, your left hand crosses your right arm and is placed to the right of your right hand. After that, your left foot crosses your right leg and is placed to the right of it. Simultaneously your right hand crosses your left arm and is placed to the right of your left hand. After each step either your arms or your legs are crossed. Do the task in a slow and concentrated manner. As soon as you move a limb that does not have its turn, you fail the task. The aim of the task is to complete 10 steps in this pattern. I’m going to count for you. Please go into the initial position. You start with your right foot and your left hand. |
| x |  | 11 | Jumping pattern | | The aim of this task is to jump in a specific pattern while rotating your arms in backward circles. You stand with your arms straight behind your back, then you make a step with one leg, jump in the air with this leg while lifting the knee of your other leg. Your arms move upwards together with your free leg. Then you land on the leg you jumped from and make a step with the leg, repeating the same pattern. You complete the task when you manage to do 5 jumps of this pattern in a row. |
| x |  | 12 | Catch a ball between the knees | | In the initial position, you bend forward, holding the ball between your knees with both hands. One hand is holding the ball from the front and the other hand is holding the ball from the back. In this position, your arms are placed around one of your legs. The aim of the test is to let go of the ball and switch the position of your hands without dropping the ball on the ground. The hand that held the ball from the front should then be holding it from the back and the hand that held the ball from the back is holding it from the front. Now, your arms are placed around your other leg. You complete the task if you manage to switch the position of your hands 3 times without dropping the ball. |
|  | x | 13 | cross the balance beam walking forwards | | For your starting position, place one foot on the beam and the other foot next to the beam on the ground. As soon as you’re ready, walk across the beam. At the end of the beam, you stop walking and stand safely with both feet on the beam for 3s. |
|  | x | 14 | Walk forwards, turn in the middle and walk back | | For your starting position, place one foot on the beam and the other foot next to the beam on the ground. As soon as you’re ready, you start walking across the beam. As soon as you crossed the middle line, you turn 180° and walk back the way you came from. At the end of the beam, you stop walking and stand safely with both feet on the beam for 3s. |
| x | x | 15 | Walk backwards, turn in the middle, walk forwards | | For your starting position, stand with your back to the beam, placing one foot on the beam and the other foot next to the beam on the ground. As soon as you’re ready, you start walking backwards. When you crossed the middle line, turn 180° and continue walking forwards to the other end of the beam. At the end of the beam, you stop walking and stand safely with both feet on the beam for 3s. |
|  | x | 16 | Walk backwards, turn 360° in the middle, continue walking backwards | | For your starting position, stand with your back to the beam, placing one foot on the beam and the other foot next to the beam on the ground. As soon as you’re ready, you start walking backwards. When you crossed the middle line, turn 360° and continue walking backward to the other end of the beam. At the end of the beam, you stop walking and stand safely with both feet on the beam for 3s. |
| x | x | 17 | Cross the beam walking forwards while dribbling a volleyball | | You may try dribbling this ball prior to the following task. For your starting position, with the volleyball in your hands, place one foot on the beam and the other foot next to the beam on the ground. As soon as you’re ready, you start dribbling the ball. Then you walk across the beam to the other end while dribbling. At the end of the beam, you stop walking and stand safely with both feet on the beam for 3s. |
|  | x | 18 | Walking forwards with eyes closed | | For your starting position, place one foot on the beam and the other foot next to the beam on the ground. As soon as you’re ready, you close your eyes and walk across the beam. You may touch the ground slightly while searching for the beam with your feet. At the end of the beam, you stop walking and stand safely with both feet on the beam for 3s. |
| x |  | 19 | Throw and catch a ball | | You stand behind this line with the ball in your hands. The aim of this task is to throw the ball with one hand against this target mark and to catch it before it hits the ground. To catch the ball you may step across the line. |
| (x) |  | 20 | Throw the ball, turn 360°, catch the ball | | You stand with the ball in your hand. As soon as you’re ready, you throw the ball into the air, turn 360° and catch the ball before it hits the ground.  (We omitted this task for safety reasons.) |

1. While the original balance test is comprised of 12 tasks, we included eyes-open conditions for two tasks involving only eyes-closed conditions (jumping jack and moving a foot around two poles in the figure of eight). We chose this procedure for safety reasons, as eyes-closed conditions are very difficult for some MS patients. [↑](#footnote-ref-2)
2. In tasks involving one-legged stances, participants were instructed to stand on the leg which in their opinion was best capable to complete the task successfully. We chose this method for economic reasons: First, using only one leg for all tasks might have induced motor fatigue in some participants. Second, testing both legs for each task would have taken too much time. [↑](#footnote-ref-3)
